# Improvement in local vascular perfusion of the lower extremities on intravoxel incoherent motion imaging: A case report

**DOI:** 10.1016/j.radcr.2022.08.029

**Published:** 2022-09-15

**Authors:** Mitsunari Maruyama, Hiroya Aso, Hisatoshi Araki, Rika Yoshida, Shinji Ando, Megumi Nakamura, Takeshi Yoshizako

**Affiliations:** Department of Radiology, Shimane University Faculty of Medicine, P.O. Box 00693-8501, 89-1 Enya cho, Izumo, Japan

**Keywords:** Intravoxel incoherent motion, Lower extremity arterial disease, Endovascular therapy

## Abstract

Intravoxel incoherent motion imaging has its improvement-evaluating ability in lower limb perfusion after endovascular therapy in individuals with lower extremity arterial disease. Here, we present a 70-year-old man with intermittent claudication of the left lower limb, whose microperfusion on intravoxel incoherent motion imaging improved after endovascular therapy. The patient underwent intravoxel incoherent motion imaging of the lower extremities pre- and postendovascular therapy. After endovascular therapy, the left ankle brachial index increased from 0.46 to 1.06. The mean perfusion-related coefficient (10^−3^ mm^2^/s) of the left lower limb increased from 19.70 ± 3.17 to 24.81 ± 3.41, and mean perfusion fraction (%) of the left lower limb slightly increased from 24.41 ± 0.96% to 25.20 ± 1.89% after endovascular therapy. Therefore, successful revascularization can improve microperfusion on intravoxel incoherent motion imaging in a patient with lower extremity arterial disease.

## Introduction

Patients with lower extremity arterial disease undergo examinations such as the ankle-brachial index test, magnetic resonance angiography (MRA), and computed tomography angiography (CTA). CTA and MRA are performed routinely to assess the distribution of stenoses or occlusions to plan the revascularization approach and to verify artery patency after treatment. However, these methods cannot evaluate improvement in microvascular perfusion of the extremities. By evaluating the perfusion, it is possible to grasp whether the perfusion is increased by endovascular therapy (EVT) and whether the wound healing is expected after EVT in chronic limb-threatening ischemia patients, which is useful for deciding the next treatment policy.

Intravoxel incoherent motion (IVIM) imaging can evaluate perfusion and diffusion, simultaneously [1]. IVIM is the microscopic translation of water molecules generated per image voxel on magnetic resonance imaging (MRI). These movements include water diffusion and microcirculation (perfusion) in tissue capillaries. When the diffusion-weighted imaging (DWI) signal is fitted to the IVIM model's advanced equation, the true diffusion coefficient (D), perfusion-related coefficient (D*), and perfusion fraction (f) are calculated via biexponential fitting analysis. IVIM is often evaluated on oncologic imaging [2], [3], [4], [5], [6]. However, IVIM imaging has not been widely used to evaluate limb perfusion [7]. In addition, IVIM imaging with the simplified IVIM techniques on 3T MRI to assess lower limb perfusion in a few minutes has not been reported.

Here, we report a case of intermittent claudication of the left lower limb, whose microperfusion on IVIM imaging improved after EVT.

## Case report

### History

A 70-year-old man presented with intermittent claudication of the left lower limb (Rutherford category 3). The initial CTA determined the location of atherosclerotic lesions that could be effectively treated with endovascular revascularization. Results showed left superficial femoral artery occlusion, categorized into type A based on the TransAtlantic InterSociety Consensus Ⅱ classification system and stage Ⅰ (FP1IP0) according to the Global Limb Anatomical Staging System classification system.

### EVT procedure

**Fig. 1:**
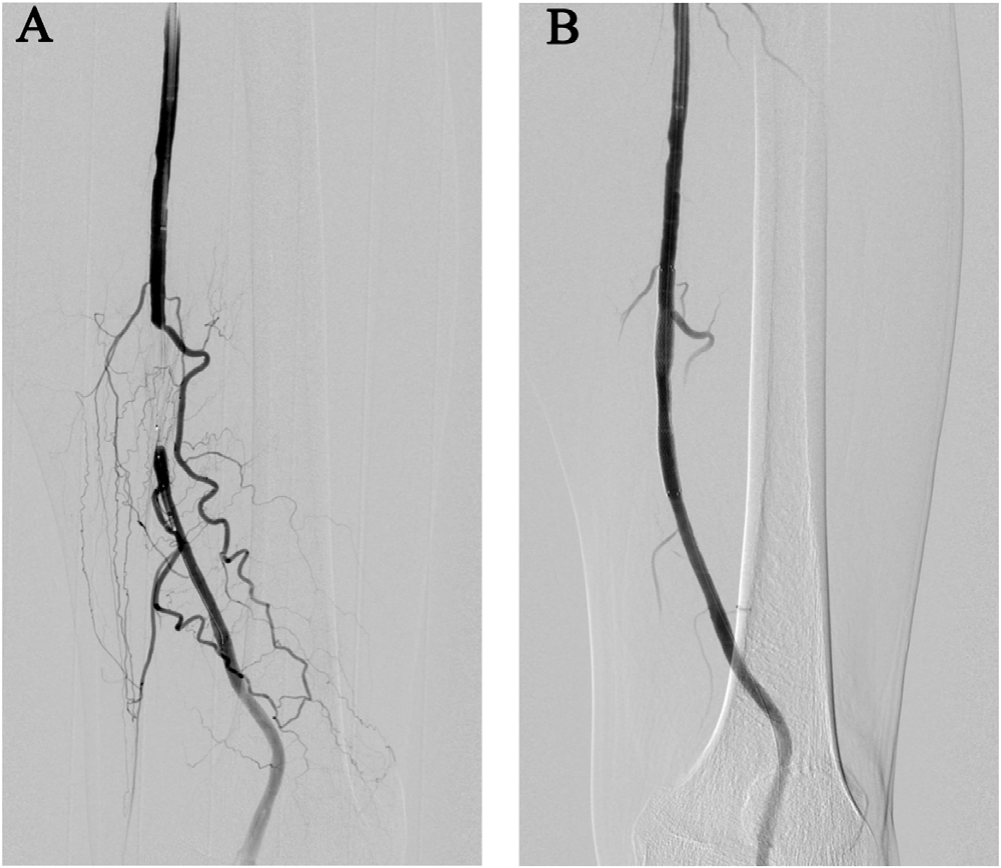
Left SFA occlusion and stent deployment. (A) DSA. Left SFA occlusion was observed. Stent deployment was performed using a fluoropolymer-coated paclitaxel-eluting stent (Eluvia; Boston Scientific Japan, Tokyo, Japan). (B) DSA after endovascular therapy. Revascularization in the left SFA was revealed. SFA, superficial femoral artery; DSA, digital subtraction angiography.

Vascular access was gained percutaneously by establishing an antegrade left common femoral artery puncture. Left superficial femoral artery occlusion was observed on digital subtraction angiography (Fig. 1A). Vessel preparation was performed using a balloon (Sterling; Boston Scientific Japan, Tokyo, Japan), and stent deployment was conducted using a fluoropolymer-coated paclitaxel-eluting stent (Eluvia; Boston Scientific Japan, Tokyo, Japan). Fig. 1B shows the digital subtraction angiography images after EVT. Significant hemodynamic improvement was noted after EVT, and the left ankle-brachial index increased from 0.46 to 1.06. Intermittent claudication of the left lower extremity (Rutherford category 3) disappeared after EVT.

### IVIM imaging

The patient underwent lower extremity IVIM imaging prior to and 2 days after EVT. Further, 3T MRI (Ingenia Elition X, Philips Medical Systems, Best, the Netherlands) was performed. Signal excitation and detection were performed using a standard quadrature RF birdcage body coil and a 20-channel head/neck coil, respectively. The conventional qualitative imaging protocol included a short-tau inversion recovery (STIR) sequence (repetition time = 5207 ms, echo time = 65 ms, inversion time = 220 ms), obtained in axial planes at the lower extremities to detect edematous changes due to infection or osteomyelitis. In total, 25 consecutive 3.5-mm thick slices and an in-plane spatial resolution (pixel size) of 0.97 × 1.97 mm^2^ (field of view = 280 × 280 mm, matrix size = 288 × 142, and reconstruction matrix = 288 × 512) were obtained using sequences. The pixel bandwidth was 516.7 Hz/pixel. The acquisition time of STIR sequence was about 1 min. For IVIM estimation, turbo spin-echo DWI with STIR lower limb axial images were acquired using different b-values (0, 300, and 1000) as follows: number of slices = 25, repetition time = 7461 ms, echo time = 130 ms, inversion time = 250 ms, flip angle = 90°, field of view = 280  × 280, matrix size = 135 × 133, and slice thickness = 3.5 mm. DWI with STIR allows a robust and more homogenous fat suppression, resulting in improved sensitivity to magnetic field inconsistencies, improved contrast-to-noise ratio, and reduced image distortion [8]. In addition, this study has used the subsecond diffusion-sensitive MRI with the modified fast spin-echo acquisition technique [9]. The DWI with STIR examinations, which lasted about 3 minutes, were conducted at a stable room temperature set at 24°C.

### IVIM imaging analysis

We performed IVIM imaging with the simplified IVIM techniques using an image analysis system (SYNAPSE VINCENT; Fujifilm Medical, Tokyo, Japan), and used the standard IVIM 2-compartment diffusion model, which included capillary perfusion and nonvascular compartments. The following biexponential equation was used to estimate signal decay:

$$\frac{SI}{SI0}=\left(1-f\right)\cdot \exp\left(-b\cdot D\right)+f\cdot \exp\left[-b\cdot \left(D+D*\right)\right],$$

where D is the true diffusion coefficient and D* is the perfusion-related coefficient. The capillary blood velocity is a factor in determining D* [10]. For each b-value, there is an associated signal intensity (SI), a zero-level signal intensity (SI0) at b = 0 s/mm^2^, and a perfusion fraction (f). The measured signal at b = 0 s/mm^2^ was used to set the SI0. At selected b-values (0, 300, and 1000 s/mm^2^), the straight line slope passing through 2 logarithmic SIs at b-values of 300 and 1000 s/mm^2^ is equivalent to D. Further, f is the difference between the line intercept and SI0. D* was obtained with the Levenberg-Marquardt method using the above biexponential equation on an image analysis system (SYNAPSE VINCENT; Fujifilm Medical, Tokyo, Japan). On each of the 25 slices, the areas of interest were set around the entire foot or distal lower leg. The mean D* (10^−3^ mm^2^/s) and f (%) were calculated pre- and post-EVT.

### Improvement in D* and f after EVT

**Fig. 2:**
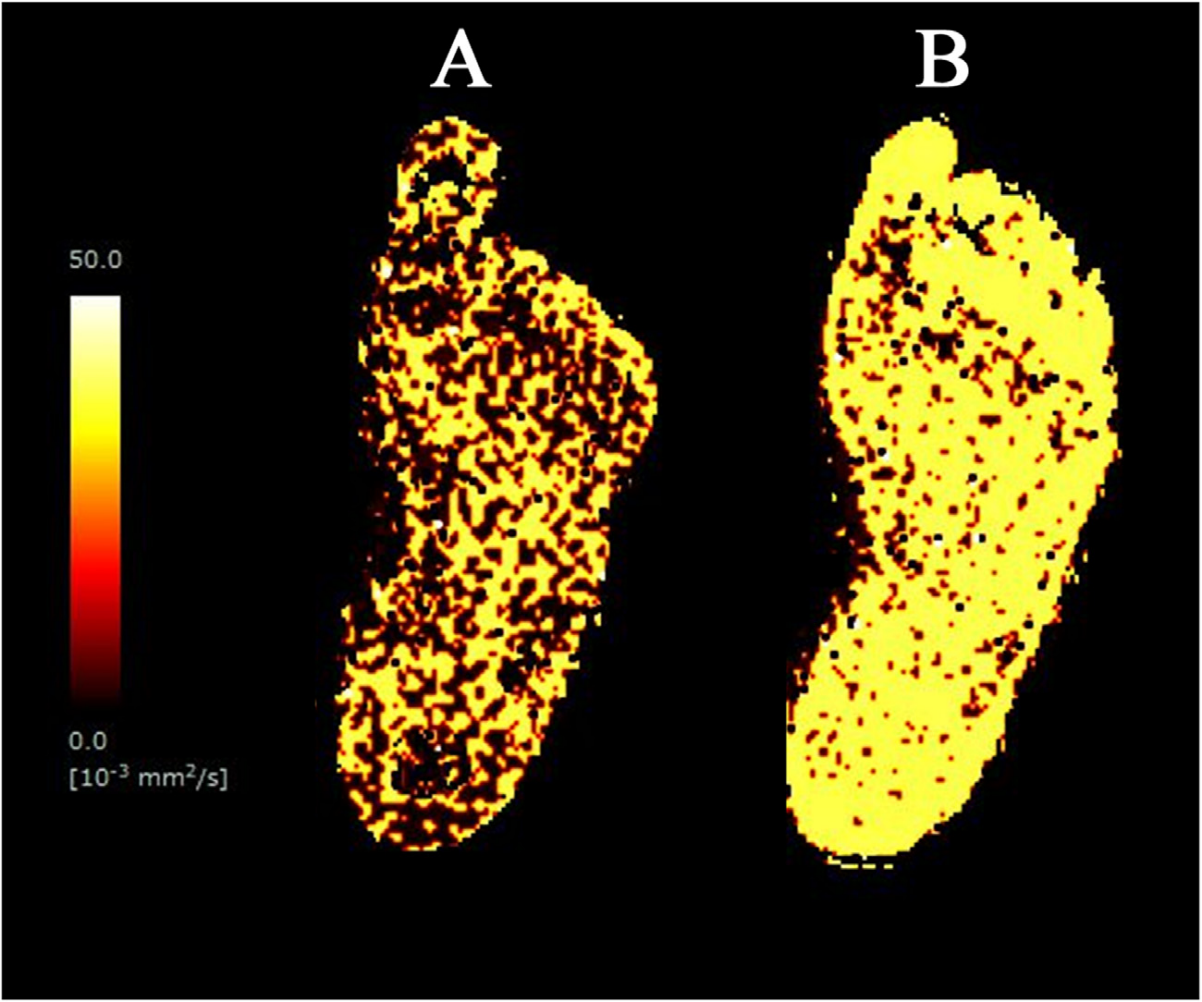
D*maps before and after EVT. (A) D*map before EVT. (B) D*map after EVT. The improvement in perfusion was revealed. D*, perfusion-related coefficient; EVT, endovascular therapy.

After EVT, mean D*(10^−3^ mm^2^/s) of left lower limb increased from 19.70 ± 3.17 to 24.81 ± 3.41 (Fig. 2, Table 1). Mean f (%) of the left lower limb slightly increased after EVT from 24.41 ± 0.96% to 25.20 ± 1.89%. Therefore, successful revascularization could improve microperfusion on IVIM imaging in our patient who presented with lower extremity arterial disease.

**Table 1:** Improvement in D* and f (%) after EVT

	Left lower limb	
	Pre	Post
Mean D* (10^−3 m^m^2^/s)	19.70 ± 3.17	24.81 ± 3.41
Mean f (%)	24.41 ± 0.96	25.20 ± 1.89
ABI	0.46	1.06

ABI, ankle-brachial index; EVT, endovascular therapy; D*, perfusion-related coefficient; f (%), perfusion fraction.

Mean D* and f (%) of the left lower limb increased after EVT.

## Discussion

We report an IVIM imaging with the simplified IVIM techniques on 3T MRI for lower limb perfusion assessment, which was obtained in a few minutes. Improvement in microperfusion after a successful EVT was observed on IVIM imaging. CTA and MRA can reveal revascularization of stenoses or occlusions. However, they cannot evaluate improvement in microvascular perfusion of the extremities. Transcutaneous assessments of oxygen partial pressure and skin perfusion pressure can evaluate tissue perfusion on a small area or a few skin points. However, they cannot supply anatomic information. Mean D*(10^−3^mm^2^/s) of the left lower limb increased after EVT, while mean f (%) of the left lower limb slightly increased after EVT. In the present case, there was only the intermittent claudication of the left lower limb (Rutherford category 3), and the f (%) was expected to be maintained. If there were resting pain, ulcer or necrosis (Rutherford categories 4-6), the f (%) might be low before EVT and more increase after EVT.

A simplified IVIM method based on DWI from 3 b-values ​​was used in this current case. Only 3 b-values are required to calculate the D and f values [1]. The simplified IVIM techniques for oncologic imaging have been used in previous studies [2,^11^,12], with the true diffusion component extracted using b-values ranging from 200 to 500 s/mm^2^. The D* value was obtained with the Levenberg-Marquardt method using the biexponential equation including the D and f values. The D and f values are determined by selected 3 b-values in a simplified IVIM method. Therefore, the previous studies [2,^11^,12] were referred for deciding the b-values. We used 300 s/mm^2^ as the cutoff b-value in this study. Further research is required to determine the optimal b-value to rule out the perfusion effect. In addition, reducing the number of b-values can shorten scan time and improve the clinical efficiency.

## Conclusion

We present a 70-year-old man with intermittent claudication of the left lower limb, whose microperfusion on IVIM imaging was noted to improve after EVT. Thus, we can conclude that IVIM imaging can evaluate improvement in lower limb perfusion after EVT.

## Patient consent

Informed consent was obtained for the publication of this case report.

## Author contributions

All authors provided substantial contributions to the manuscript and approved the final version of the article to be published.
